# AI Act Compliance Within the MyHealth@EU Framework: Tutorial

**DOI:** 10.2196/81184

**Published:** 2025-11-10

**Authors:** Monika Simjanoska Misheva, Dragan Shahpaski, Jovana Dobreva, Djansel Bukovec, Blagojche Gjorgjioski, Marjan Nikolov, Dalibor Frtunikj, Petre Lameski, Azir Aliu, Kostadin Mishev, Matjaž Gams

**Affiliations:** 1 Faculty of Computer Science and Engineering, Saints Cyril and Methodius University of Skopje, Rugjer Boshkovikj 16, Skopje, 1000, North Macedonia, 0038976472195; 2 Sorsix, Skopje, North Macedonia; 3 Zan Mitrev Clinic, Skopje, North Macedonia; 4 Adagon, Skopje, North Macedonia; 5 Ministry of Health, Skopje, North Macedonia; 6 Jožef Stefan Institute, Ljubljana, Slovenia

**Keywords:** AI Act, MyHealth@EU, OpenNCP, HL7 CDA, HL7 FHIR, Fast Healthcare Interoperability Resources, generative AI, interoperability, trustworthiness, reliability, compliance

## Abstract

The integration of artificial intelligence (AI) into clinical workflows is advancing even before full compliance with the European Union Cross-Border eHealth Network (MyHealth@EU) framework is achieved. While AI-based clinical decision support systems are automatically classified as high risk under the European Union’s AI Act, cross-border health data exchange must also satisfy MyHealth@EU interoperability requirements. This creates a dual-compliance challenge: vertical safety and ethics controls mandated by the AI Act and horizontal semantic transport requirements enforced through Open National Contact Point (OpenNCP) gateways, many of which are still maturing toward production readiness. This paper provides a practical, phase-oriented tutorial that enables developers and providers to embed AI Act safeguards before approaching MyHealth@EU interoperability tests. The goal is to show how AI-specific metadata can be included in the Health Level Seven International Clinical Document Architecture and Fast Healthcare Interoperability Resources messages without disrupting standard structures, ensuring both compliance and trustworthiness in AI-assisted clinical decisions. We systematically analyzed Regulation (EU) 2024/1689 (AI Act) and the OpenNCP technical specifications, extracting a harmonized set of overlapping obligations. The AI Act provisions on transparency, provenance, and robustness are mapped directly onto MyHealth@EU workflows, identifying the points where outgoing messages must record AI involvement, log provenance, and trigger validation. To operationalize this mapping, we propose a minimal extension set, covering AI contribution status, rationale, risk classification, and Annex IV documentation links, together with a phase-based compliance checklist that aligns AI Act controls with MyHealth@EU conformance steps. A simulated International Patient Summary transmission demonstrates how Clinical Document Architecture/Fast Healthcare Interoperability Resources extensions can annotate AI involvement, how OpenNCP processes such enriched payloads, and how clinicians in another member state view the result with backward compatibility preserved. We expand on security considerations (eg, Open Worldwide Application Security Project generative AI risks such as prompt injection and adversarial inputs), continuous postmarket risk assessment, monitoring, and alignment with MyHealth@EU’s incident aggregation system. Limitations reflect the immaturity of current infrastructures and regulations, with real-world validation pending the rollout of key dependencies. AI-enabled clinical software succeeds only when AI Act safeguards and MyHealth@EU interoperability rules are engineered together from *day 0*. This tutorial provides developers with a forward-looking blueprint that reduces duplication of effort, streamlines conformance testing, and embeds compliance early. While the concept is still in its early phases of practice, it represents a necessary and worthwhile direction for ensuring that future AI-enabled clinical systems can meet both European Union regulatory requirements from day 1.

## Introduction

With increasing artificial intelligence (AI), and particularly generative artificial intelligence (GenAI) usage, medical software systems are rapidly evolving toward integrating AI directly into clinical workflows. This shift means many such systems will be categorized as high risk under the European Union’s (EU) AI Act, requiring strict controls over multiple dimensions, including risk management, explainability, and postmarket monitoring [1]. At the same time, the EU has built a horizontal compliance and interoperability infrastructure through the European Union Cross-Border eHealth Network (MyHealth@EU), which mandates how structured health data flow between member states, passing through the Open National Contact Point (OpenNCP) software’s security gateways, and preserving semantic integrity [2]. The result is a dual-compliance framework: the AI Act regulates the functionality, that is, what intelligent systems do with data (vertical), whereas MyHealth@EU ensures the data itself are transported and interpreted consistently (horizontal). Neglecting either axis arrests deployment and introduces regulatory compliance risk.

This dual compliance framework is increasingly relevant as only some EU member states currently operate production-ready national health systems under MyHealth@EU, whereas other member states are actively building or upgrading their digital infrastructures (and in some cases, local regulatory frameworks). However, with the accelerating deployment of GenAI systems and the European Commission’s active focus on digital health powered by AI, it is likely that many future national contact points for eHealth (NCPeHs) will prefer to integrate AI from day 1 as the number one emerging technology, well before they are certified on the interoperability layer. Therefore, there is a need to provide a mechanism that ensures AI Act compliance before messages with clinical data arrive at a nonoriginating medical facility, as specified within the MyHealth@EU framework.

This work provides step-by-step guidance that translates the AI Act requirements into a sequence of implementation actions aligned with the interoperability specifications of MyHealth@EU. It includes a concise checklist to help developers assess, document, and validate AI involvement in clinical decision-making, while ensuring secure and trustworthy data exchange through the MyHealth@EU network.

The highlights of the work presented are as follows:

- Explanation of the legal and technical foundations of both the AI Act and MyHealth@EU framework,
- Explanation of the implications of AI usage within clinical decision support systems,
- Positioning of AI Act compliance into the MyHealth@EU workflow,
- Suggestion of a minimum AI-specific extension of the Health Level Seven International (HL7) Clinical Document Architecture (CDA) R2 and HL7 Fast Healthcare Interoperability Resources (FHIR) message standards, and
- Step-by-step guidelines for assuring compliance with the high-risk requirements of the AI Act and alignment with the MyHealth@EU mechanisms where possible.

This guide targets 3 overlapping audiences. Developers and data scientists will find concrete checklists that map each obligatory AI Act duty to tractable engineering artifacts. Health care IT architects and hospital deployers will see how those artifacts feed OpenNCP conformance suites, enabling plug-and-play integration with national contact points. Regulators, notified bodies, and policy makers can use the same mapping as a “transparency lens” to verify that both safety and interoperability have been considered in the case that AI was involved in any clinical decision process.

## Related Work

As the EU AI Act [1] begins its transformative role in regulating high-risk AI applications, including medical devices, a growing body of literature has emerged offering tools, checklists, and frameworks to guide functional compliance. Sovrano et al [3] presented the most direct and legally grounded approach to vertical compliance, developing a checklist strictly derived from the AI Act’s Articles 9, 13, and 14, and Annex IV. Their framework, tested on a high-risk medical expenditure prediction model, illustrates gaps in technical documentation that could hinder conformity assessment. While rigorously aligned with the AI Act, this work excludes postmarket adaptability and patient-facing usability features. Mondal [4] introduced a life cycle–oriented compliance framework tailored to machine learning–enabled in vitro diagnostic devices. By comparing the provisions of the AI Act with the In Vitro Diagnostic Medical Devices Regulation (EU) 2017/746 and International Organization for Standardization (ISO)/International Electrotechnical Commission (IEC) standards (ie, ISO 13485 [5] and ISO 14971 [6]), this work operationalizes harmonization of these regulatory provisions as pertaining to a real-world medical product pipeline. In addition to ISO 13485 and ISO 14971, the newly released ISO/IEC 42001:2023 [7] offers a comprehensive AI-specific management system standard that aligns with the governance, transparency, and life cycle control principles enshrined in the AI Act. Organizations adopting ISO 42001 can strengthen their conformity with Articles 9, 10, 13, and 14 of the AI Act, ensuring systematic oversight of high-risk AI systems.

More conceptual models, such as AI Cards by Golpayegani et al [8], provide a modular, machine-readable framework to document AI system purpose, performance, and risk in line with AI Act requirements. The Agile Safety Plan by Myklebust et al [9] adds further depth by embedding functional safety and Conformité Européenne marking into agile pipelines using protocols such as IEC 61508 and TR 5469.

Importantly, recent studies are beginning to address also the horizontal dimension. Pereira et al [10] developed an interoperability platform for the Portuguese eHealth system directly referencing MyHealth@EU specifications, identifying CDA/FHIR transformation, consent enforcement, and electronic Identification, Authentication and Trust Services (eIDAS)–based trust anchors as compliance bottlenecks. Similarly, Solomou et al [11] outlined a national architectural model that aligns mobile health systems with MyHealth@EU requirements, with preliminary compliance checklists for OpenNCP deployment. Furthermore, the Integration of Heterogeneous Data and Evidence Towards Regulatory and Health Technology Assessment Acceptance framework presented by Hussein et al [12] explicitly models cross-border semantic validation and audit logging, showing how conformity with European Health Data Space and MyHealth@EU can be harmonized. Bruthans and Jiráková [13] have assessed the technical status of cross-border ePrescription and patient summary systems in early adopter countries, confirming the necessity for validation routines and governance layers as part of national rollout plans of these and similar systems.

Despite these promising beginnings, currently, extant works still lack a synchronized compliance methodology that fully merges the AI Act’s vertical obligations with MyHealth@EU’s horizontal controls.

## Regulatory Foundations

### The EU AI Act’s Risk-Based Framework

The AI Act is a landmark: the world’s first comprehensive and legally binding regulation of AI. The AI Act has already established concrete legal requirements and prohibitions for AI systems based on their potential risk, ensuring that regulatory strictness is proportional to potential harm. The 4 risk levels along with their limits are provided in Table 1.

**Table 1.** Definition, examples, and regulatory requirements of the AI Act’s levels of risk.

Risk level	Definition	Examples	Regulatory requirements
Unacceptable risk (banned)	AI^a^ systems are considered to pose extreme harm to individuals or society and are completely prohibited.	Subliminal behavior manipulation, exploitation of vulnerable groups, social scoring systems, untargeted biometric surveillance, emotion recognition in workplaces/schools	Banned entirely from development, deployment, or use in the EU^b^.
High risk	AI that can significantly affect health, safety, or fundamental rights.	AI in medical devices, critical infrastructure control, education and employment decision systems	A comprehensive list of mandatory requirements (discussed later).
Limited risk	AI with moderate impact, mainly requiring user transparency	Chatbots, AI-generated media (images, videos), emotion detection in entertainment	Must inform users they are interacting with AI; outputs must be clearly labeled as AI generated.
Minimal risk	AI that is posing negligible or routine risks; mostly consumer-facing tools.	Spam filters, AI in video games, recommendation systems.	No specific obligations beyond general EU laws; largely unregulated under the AI Act.

a AI: artificial intelligence.

b EU: European Union.

**Figure 1.**
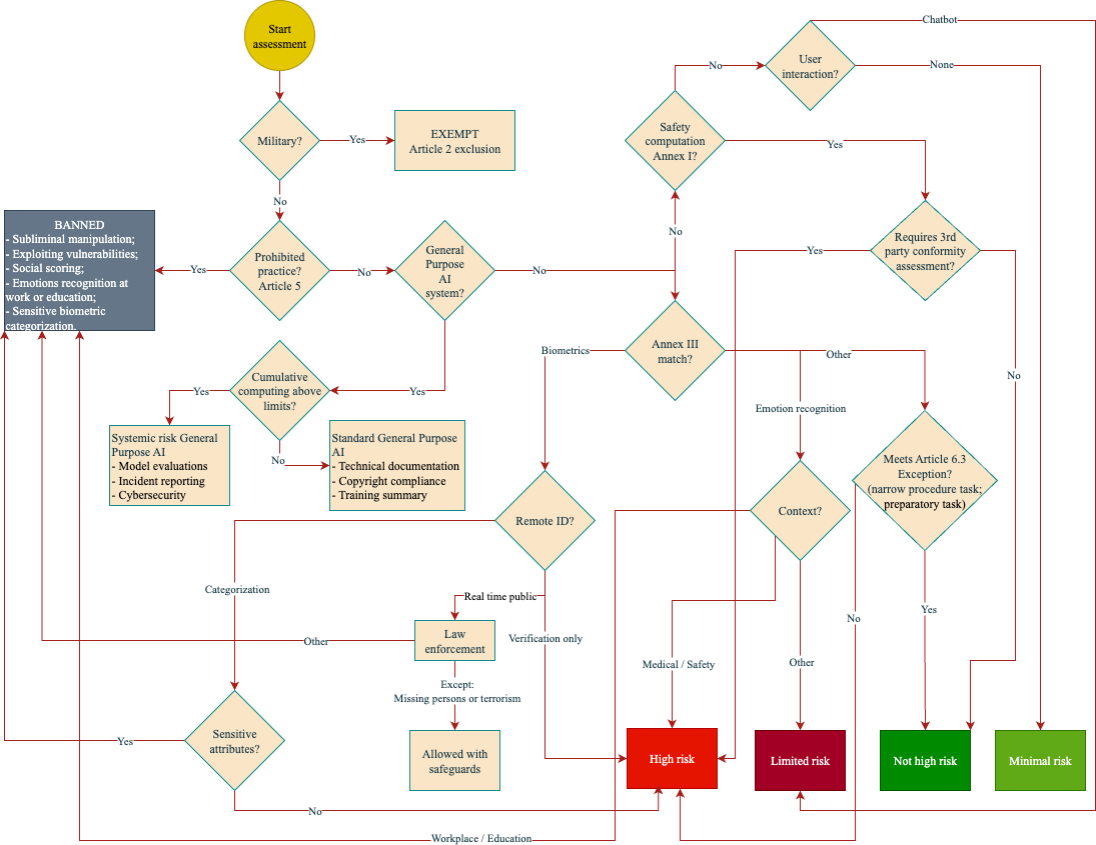
The AI Act’s level of risk assessment. AI: artificial intelligence.

To help organizations determine how the AI Act applies to their AI system, the Future of Life Institute has developed an EU AI Act Compliance Checker [14]. It is an interactive tool that guides stakeholders through a series of yes/no questions to identify the relevant obligations under the AI Act. Furthermore, the interactive checker also tailors output based on the user’s role (provider, deployer, distributor, etc), ensuring that each stakeholder knows their responsibilities. The compliance checker has been used to produce a decision flowchart (Figure 1), providing a decision tree for determining the level of risk under the AI Act, given the application domain.

Annex IV is the technical documentation regarding the AI Act, and it essentially serves as the evidence dossier for compliance, prescribing the Act’s minimum contents in detail. In general, the documentation must describe:

- the AI system’s name and version, the provider’s identity, how the system is meant to be used, the system’s overall architecture, and the input/output interactions;
- the model’s algorithmic approach, the rationale behind it, or any design trade-offs made to comply with the Act’s requirements;
- the source and characteristics of the datasets, their provenance, how they were collected and selected, and any preprocessing that was done;
- the performance measures achieved during testing and clarify the conditions under which these were obtained;
- the risk assessment process, along with the safety measures and mitigations implemented for each;
- how the concept of human-in-the-loop was enabled;
- any relevant changes made to the AI system through its life cycle and how they ensured each new version remains compliant;
- the internal control measures or best practices were used instead to achieve compliance; and
- how the provider will monitor the AI system’s performance once it is deployed.

In addition, the technical file should append a copy of the EU Declaration of Conformity (Annex V) for the AI system. This is the formal statement in which the provider declares that the system conforms to the Act (and is typically signed by an authorized person). This technical documentation is a living set of documents that must be kept up to date (at least 10 years after deployment) and made available to authorities upon request. It forces providers to deeply give consideration to and record every aspect of how the conformant AI was built and how it operates safely.

### MyHealth@EU Interoperability Framework

MyHealth@EU is the EU’s operational infrastructure for cross-border digital health data exchange. Developed under the Connecting Europe Facility’s eHealth Digital Service Infrastructure, it currently supports protocols for the routine transfer of ePrescriptions, International Patient Summaries (IPS), and original clinical documents among member states. Regulation (EU) 2025/327 formalizes common specifications for this network and outlines expansion to additional datasets, thus providing legal certainty for member states and vendors [15]. Up to date, there are 6 main dataset services, planned to act as live clinical payloads flowing through the MyHealth@EU network, that handle real patient data in production. Parts of them are fully operational (ePrescription/eDispensation, IPS, and original clinical documents); one is in a pilot stage (laboratory results), and the others are planned (medical imaging and hospital discharge report).

OpenNCP [16] is an open-source implementation of the software components that are needed to successfully run NCPeH under the semantic and technical frameworks published as under eHealth Digital Service Infrastructure. OpenNCP was originally developed under the European Patients Smart Open Services project, with 25 participating countries and over 50 beneficiaries, running from 2008 to 2014, and since 2014, it has been hosted by the European Commission.

The cross-border eHealth exchange is governed through a model that cleanly separates policy-making, technical coordination, and national implementation. Health ministry representatives (the eHealth Network), together with the European Commission’s Directorate General for Health and Food Safety, comprise the policy-making that sets the strategic agenda. They issue EU-level decisions that add new clinical domains. The same bodies ratify the semantic assets that make data interoperable, as well as approve the common cybersecurity baseline, and define how member states must report incidents.

Operated by the European Commission, the Central Services Platform (CSP) acts as the technical service hub. Through its Service Metadata Publisher (SMP), the CSP distributes an up-to-date directory of NCPeH end points and their supported transactions. A semantic repository stores and versions all CDA guides, code lists, and FHIR profiles. The CSP also runs the conformance test service, which verifies transport security, semantic validity, and workflow scenarios. The aggregated incident report confirms that each new or upgraded NCPeH meets EU requirements.

Each member state must operate at least one NCPeH instance with OpenNCP installed as the reference software, implementing the secure message exchange, template validation, consent enforcement, and auditing for every one of the MyHealth@EU dataset services. The dataset service defines what data move and how they are structured. It is the formal, standard-based data exchange capability (eg, IPS) delivered by MyHealth@EU, intended to ensure that clinicians see accurate, structured, and trusted information across borders. The core components of the OpenNCP include a Gateway service that handles AS4 messaging and mutual Transport Layer Security handshakes, a pivot transformer that maps national data models to European Union CDA templates, a security module that validates eIDAS tokens and eXtensible Access Control Markup Language (XACML) consent assertions, and an audit repository conforming to Integrating the Healthcare Enterprise (IHE) Audit Trail and Node Authentication (ATNA) profile. The project publishes binary distributions and Docker images on a regular release cycle; each aligned with the semantic-asset versions distributed by the CSP. Member states may extend OpenNCP via plug-ins provided all extensions pass the CSP Conformance Test Service. Together, these layers enable any clinician in one member state to request and receive a patient’s records from another under a single, shared rule set.

**Figure 2.**
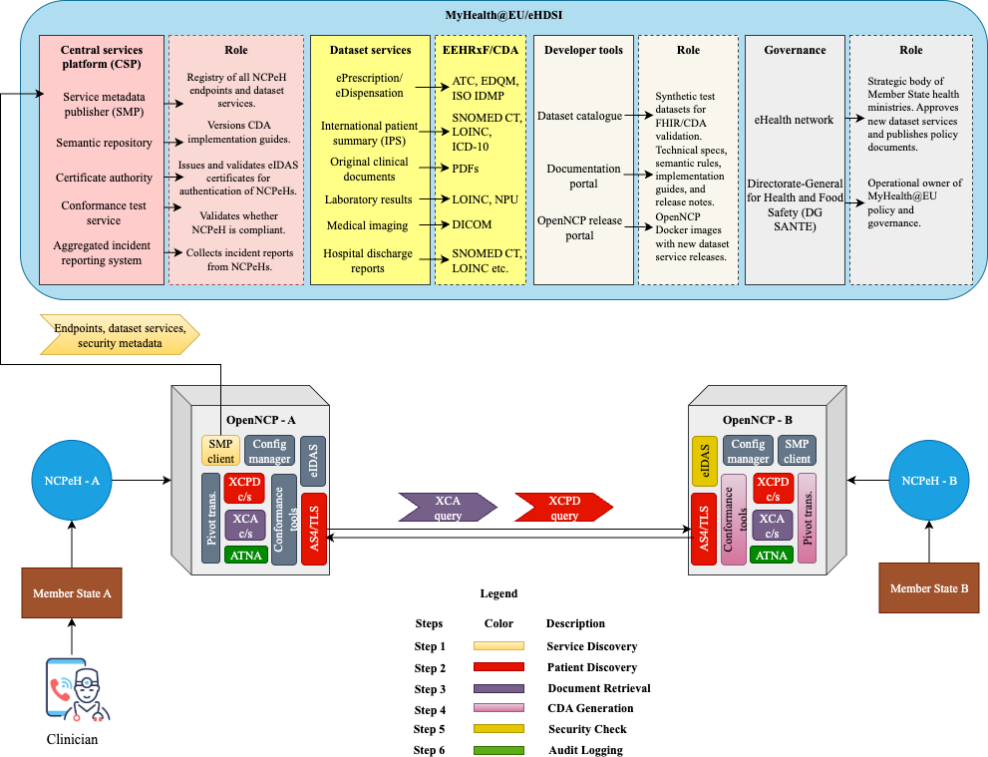
Interoperable exchange of eHealth information among member states. ASK/TLS: Asynchronous Key Transport/Transport Layer Security; ATC: Anatomical Therapeutic Chemical Classification System; ATNA: Audit Trail and Node Authentication; CDA: Clinical Document Architecture; CSP: Central Services Platform; DG SANTE: Directorate-General for Health and Food Safety; DICOM: Digital Imaging and Communications in Medicine; EDQM: European Directorate for the Quality of Medicines & HealthCare; EEHRxF: European Electronic Health Record Exchange Format; eIDAS: Electronic Identification, Authentication and Trust Services; EHR: electronic health record; FHIR: Fast Healthcare Interoperability Resources; HL7: Health Level Seven International; *ICD-10*: *International Classification of Diseases, 10th Revision*; IPS: International Patient Summary; LOINC: Logical Observation Identifiers Names and Codes; NCPeH: National Contact Point for eHealth; NPU: Nomenclature for Properties and Units; OpenNCP: Open National Contact Point (reference implementation for NCPeH); SMP: Service Metadata Publisher; SNOMED CT: Systematized Nomenclature of Medicine–Clinical Terms; TLS: Transport Layer Security; XCA: Cross-Community Access (Integrating the Healthcare Enterprise profile for document exchange); XCPD: Cross-Community Patient Discovery (Integrating the Healthcare Enterprise profile for patient identification).

Figure 2 shows an example where a clinician from member state A is requesting an IPS from member state B. It illustrates the sequence of steps, where the actors involved are marked with a corresponding color. The following process is used. Step 1 (service discovery) involves OpenNCP-A initiating a query to the SMP hosted by the CSP. Through this request, it retrieves essential interoperability metadata such as the end point address of NCPeH-B, the list of supported data set services, and relevant security certificates. Once the end point is confirmed, step 2 (patient discovery) follows. Here, OpenNCP-A’s Cross-Community Patient Discovery Client sends a patient discovery request, including demographic information, to OpenNCP-B, which uses its national master index or electronic health record (EHR) system to locate the matching individual. On successful identification, step 3 (document retrieval) begins as OpenNCP-A’s XCA client sends a formal request for the IPS to OpenNCP-B’s XCA server, which responds with the patient’s structured CDA document. Then in Step 4 (CDA generation), NCPeH-B leverages its pivot transformer to convert local EHR data into the standardized IPS CDA format. This is then semantically validated using controlled terminologies, such as Systematized Nomenclature of Medicine, Logical Observation Identifiers Names and Codes, and Unified Code for Units of Measure, and checked against Schematron rules. Then in step 5 (security and consent check), the Security Module in OpenNCP-B validates the sender’s eIDAS identity token, applies XACML-based consent policies, and securely signs and encrypts the response. With the data packaged and ready, step 6 (audit logging) is executed by both NCPeHs. Each system records detailed logs, including transaction identifiers, patient IDs, document type, and timestamps, storing these in IHE ATNA-compliant audit repositories for a minimum of 10 years.

## Implications for AI Modules Within the Clinical Decision Support Systems

AI-enabled clinical ecosystems, increasingly structured as multiagent systems with specialized roles (eg, triage, summarization, translation, and guideline validation), pose significant compliance and interoperability challenges under MyHealth@EU standards. Although the European Health Data Space regulation [15] does not yet provide explicit guidance on AI or large language models, it does require that any high-risk AI system be fully compliant with the AI Act. Ensuring transparency, provenance, and robustness—key requirements derived from the AI Act—is imperative during cross-border exchanges of clinical AI-generated or AI-handled data.

*Transparency* mandates explicit disclosure when AI generates or transforms clinical data, clearly indicating the AI model, assumptions, and oversight involved. For example, AI-generated narrative summaries must annotate the contribution of AI clearly, fulfilling AI Act transparency obligations.

*Provenance* expands transparency by thoroughly documenting data origin, transformation history, model versions, timestamps, national identifiers, and reviewer credentials. This comprehensive metadata enables clinicians to verify data integrity and trustworthiness, addressing the Act’s data governance and technical documentation mandates.

*Robustness* safeguards schema integrity, controls terminology usage, secures multiagent data exchanges, and adheres to audit logging standards. It aligns with AI Act provisions on risk management, cybersecurity, human oversight, and postmarket monitoring.

## Walkthrough

### Embedding EU AI Act Compliance Within the MyHealth@EU Framework

**Figure 3.**
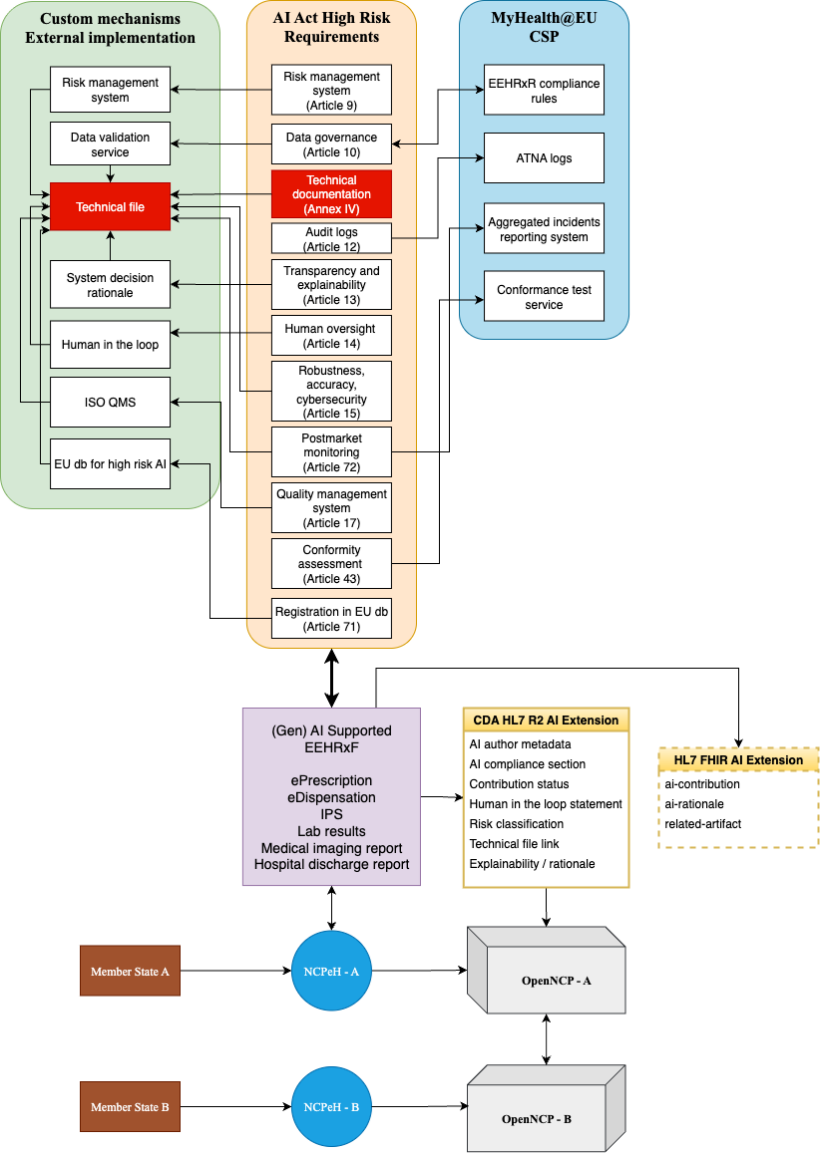
The AI Act position within the MyHealth@EU framework. AI: artificial intelligence; ATNA: Audit Trail and Node Authentication; CDA: Clinical Document Architecture; CSP: Central Services Platform; EEHRxF: European Electronic Health Record Exchange Format; EU: European Union; FHIR: Fast Healthcare Interoperability Resources; HL7: Health Level Seven International; IPS: International Patient Summary; ISO: International Organization for Standardization; MyHealth@EU: European Union Cross-Border eHealth Network; NCPeH: National Contact Point for eHealth; OpenNCP: Open National Contact Point; QMS: quality management system.

Member states are expected to incorporate AI technologies into their national health care systems, necessitating adherence to the EU AI Act without imposing modifications at the MyHealth@EU infrastructural level. Figure 3 positions an AI-based system within a hypothetical national health care system in member state A. All AI-related regulatory requirements must be addressed before interfacing the national EHR system with the OpenNCP. Specifically, high-risk AI systems under the AI Act must comply with requirements for risk management, data governance, technical documentation, audit logging, transparency and explainability, human oversight, robustness, postmarket monitoring, quality management systems, conformity assessment, and registration in an EU database.

Analysis of these requirements indicates that some can benefit from existing MyHealth@EU policies, whereas others necessitate external solutions provided by developers responsible for their reliability. Central to these requirements is the technical documentation mandated by Annex IV of the AI Act, serving as a comprehensive reference repository accessible via URL. This documentation, stored securely alongside external services, provides essential details about the AI system.

Risk management, lacking direct equivalents within MyHealth@EU, must be addressed externally by clearly outlining the risk analysis life cycle, mitigation strategies, hazard modeling, residual risk management, and continuous risk monitoring procedures. Data governance, critical for AI-driven processes, also requires external oversight to manage dataset origin, representativeness, preprocessing techniques, and bias mitigation strategies, guided by compliance rules derived from European Electronic Health Record Exchange Format standards pertinent to medical data.

Audit logs, essential for system traceability, must adhere to the IHE ATNA profile, ensuring transparent documentation of system operations. Transparency and explainability pose significant challenges within AI applications and necessitate external mechanisms thoroughly detailed in the technical documentation. Human oversight mechanisms must clearly define clinical responsibilities, supervisory roles, decision reviews, and emergency override protocols, incorporating clinical corrections into documentation.

Robustness, accuracy, and cybersecurity documentation should provide comprehensive evidence of system reliability and safety. This includes detailed performance metrics, stress testing results under edge-case clinical conditions, error handling procedures, adversarial resistance evaluations, and security-by-design mechanisms. For generative AI and multiagent systems, developers should consult security guidelines, such as those outlined by the Open Worldwide Application Security Project (OWASP) GenAI Security Project [17], which highlight critical risks such as prompt injection, model extraction, and misuse, offering mitigation strategies tailored to large language model–based architectures.

Postmarket monitoring must define clear frameworks for detecting performance drift, integrating user feedback into iterative updates, and managing incidents effectively. This also entails alignment with the MyHealth@EU incident reporting and aggregation system, ensuring timely and standardized communication of serious events across member states.

Quality management systems should be implemented in accordance with internationally recognized standards. In particular, ISO/IEC 42001 provides a dedicated framework for auditing, certifying, and governing AI management systems. As Benraouane [18] emphasizes, this standard is instrumental in embedding risk-based thinking, traceability, and accountability into AI product life cycle management, the elements that are foundational for both regulatory compliance and ethical assurance in clinical environments.

The CSP Conformance Testing Service acts as the final validation point, verifying compliance with document formats, security and transport envelopes, and audit trail integrity. Finally, the registration of high-risk AI systems in an EU database remains under regulatory consideration.

Fulfillment of these criteria allows AI-generated content, such as IPSs, ePrescription/eDispensation documents, discharge reports, laboratory results, and other future MyHealth@EU services, to enrich standardized clinical documents. These documents are typically formatted using either the HL7 CDA, a widely used XML-based format, or the future HL7 FHIR.

Tables2  3 present an effort to incorporate a minimal AI Compliance subsection within the HL7 CDA and HL7 FHIR standards, correspondingly, without compromising the standard fields, including:

- *AI author metadata*: detailing AI engine specifics
- *AI compliance section*: isolated container for AI-exclusive data points
- *Contribution status*: machine-readable indication of AI-generated content
- *Human-in-the-loop statement:* documentation of human oversight
- *Risk classification*: designation of risk level for monitoring purposes
- *Technical-file link*: direct URL access to Annex IV documentation
- *Explainability rationale*: transparent reasoning behind AI-generated decisions

**Table 2.** Health Level Seven International Clinical Document Architecture R2 core structure with minimal AI (artificial intelligence)–specific extensions.

Layer	Description	Key child elements—(*) is a repeatable element
Header	Carries document context and metadata so any system can identify who the document is about, what it is, who/what authored it, when it was created, and under whose authority it was released	typeId templateId* id code title effectiveTime confidentialityCode languageCode recordTarget author* AI author metadata (assignedAuthoringDevice) manufacturerModelName softwareName dataEnterer (optional) informant* authenticator*/legalAuthenticator custodian informationRecipient* participant* inFulfillmentOf documentationOf* relatedDocument* authorization componentOf (encounter context)
Body	Holds the clinical content (could be a free-text field or as a structured body with coded sections and entries)	Component structuredBody AI compliance section templateId (AI-section profile) code (AI-META) title text (narrative) entry Contribution status entry Human-in-the-loop statement entry Risk classification entry Technical-file link entry Explainability rationale Additional clinical section* (History, Medications, Results,...) templateId* code title text entry* (observations, acts, procedures)

**Table 3.** Health Level Seven International Fast Healthcare Interoperability Resources core structure with minimal artificial intelligence (AI)–specific extensions.

Bundle	Description	AI-specific integration
*DiagnosticReport*	Provides narrative findings and points to supporting *Observation*.	extension[ai-contribution]—AI generation status extension[ai-rationale]—explaining model reasoning extension[ai-artifact]—linking to Annex IV technical file extension[ai-risk]—risk class
*Observation*	Holds a discrete clinical data point (eg, lesion size, laboratory value).	extension[ai-contribution]—stating that values were AI-derived
*Provenance*	Records who (person or system) produced or modified the target resource and when.	Provenance.agent.extension[ai-contribution]—human verification with possible overriding original statement

Given that future HL7 FHIR implementation would contain a DiagnosticReport for the summary, Observation for a discrete finding, and Provenance to record authorship and timing, the AI-related fields should be implemented as FHIR extensions to those resources with AI contributions, rationale, documentation links, AI confidence, interpretative metrics, and agent involvement as given in Table 3.

We intentionally avoided recommending new AI-based profiles, such as AIDiagnosticReport, to avoid adding complexity while the current draft profiles are still being finalized; this choice postpones fragmentation and makes it easier to converge on a future European AI profile.

For AI Act compliance, the key requirement is transparency, being able to flag AI involvement and link to the Annex IV technical file. These optional extensions satisfy that without imposing new mandatory constraints on trading partners. These extensions would be considered optional and valid only when AI has been included in some of the clinical reports without imposing new mandatory constraints on trading partners.

### Checklist for AI-Act Compliance Before OpenNCP Transmission

Table 4 presents a step-by-step guideline, which outlines essential actions to ensure clinicians that any AI-generated decision wrapped in the data message they receive through MyHealth@EU/OpenNCP complies fully with the AI Act. The same guidelines can also be used for AI Act–compliant national transfer of the clinical data.

**Table 4.** The AI Act compliance checklist for European Union Cross-Border eHealth Network (MyHealth@EU)–ready integration.

Phase and step	Action	Description/artifacts	Status
Phase 0: Project framing			
0.1	Confirm clinical scope	Identify which dataset service the AI^a^ touches (ePrescription, IPS^b^, imaging, etc)	☐
0.2	Classify as high risk	Record legal basis (health diagnosis/treatment) in project log	☐
0.3	Setup Annex IV repo	Secure version-controlled URL that will store all compliance artifacts	☐
Phase 1: Build external AI Act controls			
1.1	Risk management system (Art 9)	Create hazard list, mitigation matrix	☐
1.2	Data governance (Art 10)	Document dataset provenance, representativeness, preprocessing and bias checks; align codes with EEHRxF^c^	☐
1.3	Technical documentation population	Populate Annex IV file set: architecture, test results, certificates	☐
1.4	Automatic logs (Art 12)	Extend ATNA^d^ to record model ID + inference timestamps	☐
1.5	Transparency/XAI^e^ (Art 13)	Generate human-readable rationale + JSON payload	☐
1.6	Human oversight (Art 14)	Deploy HITL^f^ platform with override option	☐
1.7	Robustness and security (Art 15)	Run stress tests and archive metrics. Consult security guidelines [17].	☐
1.8	Quality management system (Art 17)	Operate ISO^g^ compliant QMS^h^ (eg, ISO 13485 [5] for medical devices, ISO 42001 for AI systems [18])	☐
1.9	Conformity assessment (Art 43)	Complete internal or notified-body route; store certificate	☐
1.10	Postmarket monitoring (Art 72)	Implement drift metrics, feedback loop, incident workflow	☐
1.11	Prepare EU^i^ registration (Art 71)	Draft JSON payload for future EU AI database	☐
Phase 2: Generate AI-enriched payload			
2.1	Build CDA^j^/FHIR^k^ document	Use the correct EEHRxF template (eg, ImagingReport, IPS)	☐
2.2	Inject AI markers	Add AI-info sections (CDA) or ai-* extensions (FHIR)	☐
Phase 3: Conformance and exchange			
3.1	Schema validate locally	Use an off-the-shelf CDA/FHIR validator	☐
3.2	Wrap security envelope	Wrap the document in the same XDS^l^/XDR^m^ message your NCPeH^n^ expects	☐
3.3	Merge ATNA log entry	Create an ATNA record	☐
3.4	Run CSP^o^ Test Service	Upload the message to the CSP Test Service	☐
3.5	Transmit via OpenNCP^p^	Route the validated message through your NCPeH. OpenNCP transforms nothing; it simply forwards to member state B. Remote clinicians see a normal report, the AI block appears only in AI-aware UIs	☐
Phase 4: Operate, monitor, and evolve			
4.1	Collect postmarket monitoring metrics	Deploy a metrics collector: model latency, accuracy drift, false-positive rate	☐
4.2	Detect and report incidents	File serious-incident JSON to EU portal within 15 days. Link incident ID back into Annex IV folder	☐
4.3	Update model under QMS	Log new version of the software	☐
4.4	Refresh tech file	Replace Annex IV PDF; keep URL constant	☐
4.5	EU database registration (when portal opens)	Post JSON payload when registry goes live; embed EU AI ID in reports	☐
4.6	Annual reconformity check	Rerun CSP test on a sample message.	☐

a AI: artificial intelligence.

b IPS: International Patient Summary.

c EEXRxF: European Electronic Health Record Exchange Format.

d ATNA: Audit Trail and Node Authentication.

e XAI: explainable artificial intelligence.

f HITL: human-in-the-loop.

g ISO: International Organization for Standardization.

h QMS: Quality Management System.

i EU: European Union.

j CDA: Clinical Document Architecture.

k FHIR: Fast Healthcare Interoperability Resources.

l XDS: Cross-Enterprise Document Sharing.

m XDR: Cross-Enterprise Document Reliable Interchange.

n NCPeH: National Contact Point for eHealth.

o CSP: Central Services Platform.

p OpenNCP: Open National Contact Point.

## Illustrative Examples: A Simulation of AI-Supported IPS Transmission

To demonstrate how the proposed CDA/FHIR extensions and checklist actions can be applied in practice, we simulate an AI-supported IPS transmission between 2 member states (member state A and member state B).

### Step 1: IPS Generation With AI Contribution

Within member state A, a hospital EHR generates an IPS enriched by an AI module, which performs a summarization of discharge notes. The CDA header records the standard metadata, while the AI compliance section explicitly marks AI involvement (Textbox 1).

Textbox 1. **Health Level Seven Clinical Document Architecture (HL7 CDA) representing the International Patient Summary (IPS) enriched with AI contributions.**

<component>

<structuredBody>

<component>

<section>

<templateId root=“2.16.840.1.113883.AI-META”/>

<code code=“AI-META” displayName=“AI Compliance Metadata”/>

<title>AI Compliance Information</title>

<text>AI-assisted summarization of discharge notes</text>

<entry>

<observation classCode=“OBS” moodCode=“EVN”>

<code code=“AI-CONTRIBUTION” displayName=“AI Contribution”/>

<value xsi:type=“BL” value=“true”/>

</observation>

</entry>

<entry>

<observation>

<code code=“AI-RISK” displayName=“Risk Classification”/>

<value xsi:type=“CD” code=“high-risk”/>

</observation>

</entry>

<entry>

<observation>

<code code=“AI-RATIONALE” displayName=“Explainability Rationale”/>

<value xsi:type=“ST”>Summary generated using validated LLM model v2.3</value>

</observation>

</entry>

<entry>

<observation>

<code code=“AI-ANNEXIV” displayName=“Annex IV Technical File”/>

<value xsi:type=“URI”>https://hospital.eu/annexIV/ips-ai-summary-v2.3</value>

</observation>

</entry>

</section>

</component>

</structuredBody>

</component>

### Step 2: Equivalent Representation in HL7 FHIR

If the IPS were represented in FHIR, the AI metadata would be injected via extensions (Textbox 2).

Textbox 2. **Health Level Seven Fast Healthcare Interoperability Resources (HL7 FHIR) JSON representation of the International Patient Summary (IPS) enriched with AI contributions.**

{

“resourceType”: “DiagnosticReport”,

“id”: “ips-summary-1234”,

“status”: “final”,

“code”: { “coding”: [{ “system”: “http://loinc.org”, “code”: “60591‐5”, “display”: “Patient summary Document” }] },

“subject”: { “reference”: “Patient/123” },

“extension”: [

{ “url”: “http://hospital.eu/fhir/StructureDefinition/ai-contribution”, “valueBoolean”: true },

{ “url”: “http://hospital.eu/fhir/StructureDefinition/ai-risk”, “valueCode”: “high-risk” },

{ “url”: “http://hospital.eu/fhir/StructureDefinition/ai-rationale”, “valueString”: “Summary generated using validated LLM model v2.3” },

{ “url”: “http://hospital.eu/fhir/StructureDefinition/ai-annexIV”, “valueUri”: “https://hospital.eu/annexIV/ips-ai-summary-v2.3” }

]

}

### Step 3: Secure Transmission via OpenNCP

The following describes how data is transmitted securely.

1. Service discovery—NCPeH-A queries CSP’s SMP to locate NCPeH-B
2. Patient discovery—patient identified across member states
3. Document generation—IPS assembled with embedded AI section/extensions
4. Semantic validation—CDA/FHIR validated against EU templates, Schematron rules, and controlled terminologies (Systematized Nomenclature of Medicine, Logical Observation Identifiers Names and Codes)
5. Security and consent checks—eIDAS identity token and XACML consent validated
6. Audit logging—ATNA logs enriched with modelID and inference timestamp
7. Transmission—validated IPS sent via AS4 channel to NCPeH-B

### Step 4: Clinician View in Member State B

In member state B, the IPS is displayed in the local EHR. For clinicians using AI-aware interfaces, the “AI Compliance Information” block appears in a separate panel. For systems not supporting the extensions, the IPS remains schema-compliant and renders as a normal IPS, ensuring backward compatibility.

## Discussion

### Security Risks

AI-enabled clinical systems introduce unique vulnerabilities that go beyond conventional cybersecurity threats. While the AI Act requires “robustness, accuracy, and cybersecurity” (Art 15), practical guidance is limited. To operationalize security within the compliance checklist, the OWASP GenAI Security Project risks have been aligned with concrete safeguards and their placement in the proposed phase-oriented framework as presented in Table 5.

**Table 5.** Open Worldwide Application Security Project generative artificial intelligence (AI) security risks position within the proposed checklist.

Risk	Description	Mitigation strategy	Checklist phase
Prompt injection	Maliciously crafted input manipulates the model to override safety controls or leak sensitive data	Input sanitization and context filtering; restrict model to domain-specific prompts; log all inference inputs in ATNA^a^	Phase 1.7 (Robustness & Security)
Data poisoning	Training or fine-tuning data manipulated to bias outputs	Dataset provenance documentation; bias checks and representativeness tests; secure training pipelines	Phase 1.2 (Data Governance)
Model inversion/extraction	Attackers query models to reconstruct sensitive training data or extract model parameters	Rate-limiting queries; differential privacy techniques; deploy inference APIs^b^ behind NCPeH^c^ security gateways	Phase 1.7 (Robustness & Security)
Adversarial inputs	Subtle perturbations to inputs mislead the AI (eg, medical image modifications)	Stress testing with adversarial datasets; human-in-the-loop verification for high-impact decisions; archive adversarial test metrics	Phase 1.7 (Robustness & Security)
Misuse and abuse	Legitimate model repurposed for unintended or harmful applications.	Annex IV documentation describing intended use; continuous postmarket monitoring of deployment contexts	Phase 1.3 (Technical Documentation) and phase 4.1‐4.3 (Monitoring)

a ATNA: Audit Trail and Node Authentication.

b API: application programming interface.

c NCPeH: National Contact Point for eHealth.

### Postmarket Monitoring, Risk Assessment, and Incident Aggregation

The EU AI Act (Art 72) requires providers of high-risk AI systems to implement continuous postmarket monitoring to detect performance drift, manage incidents, conduct systematic risk assessments, and ensure that deployed models remain compliant throughout their life cycle. In parallel, MyHealth@EU mandates incident reporting and aggregation across member states, ensuring that system-wide risks are captured at the EU level. Integrating these 2 dimensions avoids fragmented monitoring, strengthens ongoing risk management, and builds clinical trust.

By mapping these dimensions side by side, developers and implementers gain a clear view of where obligations overlap, where they complement each other, and how a single workflow can satisfy both regimes. This mapping also highlights the types of artifacts (metrics, audit logs, JSON reports) that must be collected at each step to ensure traceability and trustworthiness across national borders.

Table 6 presents these monitoring dimensions, their regulatory anchors, and practical implementation measures. It demonstrates how local monitoring systems can be directly aligned with MyHealth@EU’s interoperability and incident aggregation mechanisms. Together, these measures create a unified monitoring pipeline that reduces duplication, enforces consistency, and strengthens clinical confidence in AI-enabled cross-border health data exchange.

**Table 6.** Practical implementation of monitoring demands.

Dimension	AI Act requirement	MyHealth@EU^a^ alignment	Practical implementation
Performance drift	Continuous evaluation of accuracy, robustness, and bias after deployment	Not directly mandated but affects semantic validity of exchanged data	Deploy drift detection metrics (eg, false positive rate, latency, accuracy vs baseline). Log deviations into Annex IV repository.
User feedback loops	Collect, document, and incorporate clinician/patient feedback	Can be integrated with audit and consent systems	Provide feedback forms linked to IPS^b^/ePrescription viewers; map feedback IDs to ATNA^c^ audit entries.
Serious incident detection	Incidents must be reported within 15 days to EU^d^ authorities	MyHealth@EU CSP^e^ runs an incident aggregation service across NCPeHs^f^	Trigger automatic incident JSON payload generation when system detects failures, link to Annex IV URL
Model updates	Providers must log new versions and reconduct conformity checks	Ensures updated versions still pass CSP conformance tests	Refresh Annex IV technical file and rerun conformance tests before redeployment
Security alerts	Adversarial or injection attempts must be documented	CSP incident aggregation accepts security-related events	Configure monitoring dashboards to flag OWASP^g^-related anomalies and escalate them to the CSP

a MyHealth@EU: European Union Cross-Border eHealth Network.

b IPS: International Patient Summary.

c ATNA: Audit Trail and Node Authentication.

d EU: European Union.

e CSP: Central Services Platform.

f NCPeH: National Contact Point for eHealth.

g OWASP: Open Worldwide Application Security Project.

### Limitations

While the tutorial proposes a harmonized pathway for embedding EU AI Act compliance within the MyHealth@EU interoperability framework, several limitations remain:

1. *Simulation rather than real-world validation*. The CDA/FHIR extensions and compliance checklist are demonstrated through a simulation of IPS transmission. Although this walkthrough shows technical feasibility, no production deployment has yet validated the approach in live NCPeHs. Future work will involve pilot integrations with vendor systems and hospital deployments to confirm interoperability under operational constraints.
2. *Backward compatibility and vendor diversity*. The proposed AI metadata extensions are deliberately lightweight and optional, ensuring that CDA/FHIR payloads remain schema compliant even when AI fields are ignored. However, interoperability across heterogeneous vendor systems may surface edge cases (eg, legacy CDA parsers discarding unknown sections or inconsistent FHIR extension handling). Vendor engagement and conformance testing will be required to confirm seamless adoption.
3. *Performance trade-offs*. Adding AI metadata and monitoring layers introduces overhead. Extended ATNA logging, JSON payload packaging, and continuous drift detection can increase processing latency and storage costs. In high-volume clinical settings, this may affect throughput. While the proposed extensions are minimal, performance benchmarking in operational environments is essential to quantify trade-offs.
4. *Evolving regulatory and semantic landscape*. Both the AI Act and MyHealth@EU are subject to iterative refinement. Some provisions, such as EU database registration for high-risk AI systems, are not yet operational. Similarly, upcoming expansions of MyHealth@EU datasets (eg, imaging, laboratory results) may require additional adaptations. The presented framework should therefore be seen as a blueprint aligned with the current regulatory state, but requiring updates as legal and technical specifications evolve.

These limitations are not shortcomings of the proposed framework itself but reflect the immaturity of the ecosystem as a whole. Both the EU AI Act and the MyHealth@EU framework are in their earliest implementation stages, with several provisions and datasets still under development. Technically, national infrastructures and vendor systems are not yet fully equipped to conduct real-world validation of AI-augmented interoperability. Dependencies such as the EU AI database, expanded MyHealth@EU dataset services (eg, imaging, laboratory results), and vendor readiness must mature before end-to-end validation is feasible. Until then, the tutorial provides simulated examples that anticipate these developments and prepare the ground for future pilot deployments.

### Conclusions

Deploying AI-enabled clinical decision support across Europe requires treating regulatory compliance (AI Act) and technical interoperability (MyHealth@EU) as inseparable design constraints. This tutorial has translated the legal language of the AI Act and the technical specifications of MyHealth@EU into a single, step-by-step blueprint for developers, architects, and policymakers.

By providing a phase-oriented checklist, lightweight CDA/FHIR extensions, and illustrative examples such as an AI-supported IPS transmission, we have shown how AI systems can be engineered to satisfy high-risk obligations while remaining interoperable with existing OpenNCP workflows. The tutorial also integrates emerging security practices from OWASP GenAI and aligns postmarket monitoring duties with MyHealth@EU’s incident aggregation framework.

For developers, the practical implications are clear:

- Reduced duplication of effort—one compliance path covers both the AI Act and MyHealth@EU requirements
- Smoother conformance testing—AI metadata are embedded early, ensuring messages pass CSP validation without repeated rework.
- Early compliance assurance—Annex IV documentation, audit logging, and risk management are built in “from day 0,” lowering regulatory delays.
- Backward compatibility—minimal extensions preserve schema validity, enabling adoption without disrupting existing vendor systems.

As member states expand their National Contact Points and clinical datasets, this tutorial provides a reusable blueprint for integrating AI capabilities into cross-border health care. While real-world validation remains a next step, the presented framework offers developers an actionable guide to design, implement, and monitor AI-enabled systems that are simultaneously trustworthy, interoperable, and compliant.

This work is forward-looking, anticipating the convergence of the AI Act and MyHealth@EU frameworks. Although the concept is still in its early phases to be put into practice, it offers a worthwhile blueprint that equips developers to prepare for compliance and ensures that future AI-enabled clinical systems can meet both requirements from day 1.

## Abbreviations

AI: artificial intelligence
ATNA: Audit Trail and Node Authentication
CDA: Clinical Document Architecture
CSP: Central Services Platform
eIDAS: Electronic Identification, Authentication and Trust Services
EU: European Union
FHIR: Fast Healthcare Interoperability Resources
GenAI: generative artificial intelligence
HL7: Health Level Seven International
IHE: Integrating the Healthcare Enterprise
IPS: International Patient Summary
ISO: International Organization for Standardization
MyHealth@EU: European Union Cross-Border eHealth Network
NCPeH: National Contact Point for eHealth
OpenNCP: Open National Contact Point
OWASP: Open Worldwide Application Security Project
SMP: Service Metadata Publisher
XACML: eXtensible Access Control Markup Language
